# Laparoscopic bladder injury and ascites

**DOI:** 10.4103/0972-9941.78360

**Published:** 2011

**Authors:** Viroj Wiwanitkit

**Affiliations:** Wiwanitkit House, Bangkhae, Bangkok Thailand 10160

Dear Sir,

I read the recent report on laparoscopic bladder injury-induced ascites with great interest.[1] I would like to share some ideas on this study. There are two main points to be discussed. First, the question of how to prevent laparoscopic bladder injury should be addressed. Good anatomical clarification on the surgical site is necessary for all laparoscopic surgeries. If the case is still doubtful, selection of laparotomy might be more appropriate. Nevertheless, to prevent bladder injury, Hsieh *et al*. proposed the ‘placement of a urethral catheter and syringe-assisted drainage of all urine from the bladder at the beginning of the operation’.[2] However, based on good preparation, good instrument, good surgeon and team, a bladder injury can still occur. Hence, it is necessary to re-check, intra-operatively, before making a conclusion that there is no complication. Wu *et al*. noted that ‘early recognition of injuries, preferably intra-operatively, with immediate appropriate treatment is crucial’.[3] Second, the laboratory diagnosis on urinary ascites should be discussed. Indeed, as Al-Mandeel *et al*, observed, it is not necessary that this condition be accompanied with aberrant clinical chemistry results. The diagnosis of ascites must be based on the existence of fluid in the abdominal wall and the analysis of ascetic fluid can be useful for making a definitive diagnosis.[4]
